# Novel Carbonyl Analogs of Tamoxifen: Design, Synthesis, and Biological Evaluation

**DOI:** 10.3389/fchem.2017.00071

**Published:** 2017-09-26

**Authors:** Konstantinos M. Kasiotis, George Lambrinidis, Nikolas Fokialakis, Evangelia N. Tzanetou, Emmanuel Mikros, Serkos A. Haroutounian

**Affiliations:** ^1^Laboratory of Pesticides Toxicology, Department of Pesticides Control and Phytopharmacy, Benaki Phytopathological Institute, Athens, Greece; ^2^Division of Pharmaceutical Chemistry, Department of Pharmacy, School of Health Sciences, National and Kapodistrian University of Athens, Athens, Greece; ^3^Division of Pharmacognosy and Natural Products Chemistry, Department of Pharmacy, School of Health Sciences, National and Kapodistrian University of Athens, Athens, Greece; ^4^Laboratory of Nutritional Physiology and Feeding, Faculty of Animal Sciences and Aquaculture, Agricultural University of Athens, Athens, Greece

**Keywords:** tamoxifen, synthesis, derivatives, docking, MCF-7

## Abstract

Aim of this work was to provide tamoxifen analogs with enhanced estrogen receptor (ER) binding affinity. Hence, several derivatives were prepared using an efficient triarylethylenes synthetic protocol. The novel compounds bioactivity was evaluated through the determination of their receptor binding affinity and their agonist/antagonist activity against breast cancer tissue using a MCF-7 cell-based assay. Phenyl esters **6a,b** and **8a,b** exhibited binding affinity to both ERα and ERβ higher than 4-hydroxytamoxifen while compounds **13** and **14** have shown cellular antiestrogenic activity similar to 4-hydroxytamoxifen and the known ER inhibitor ICI182,780. Theoretical calculations and molecular modeling were applied to investigate, support and explain the biological profile of the new compounds. The relevant data indicated an agreement between calculations and demonstrated biological activity allowing to extract useful structure-activity relationships. Results herein underline that modifications of tamoxifen structure still provide molecules with substantial activity, as portrayed in the inhibition of MCF-7 cells proliferation.

## Introduction

**Figure 1:**
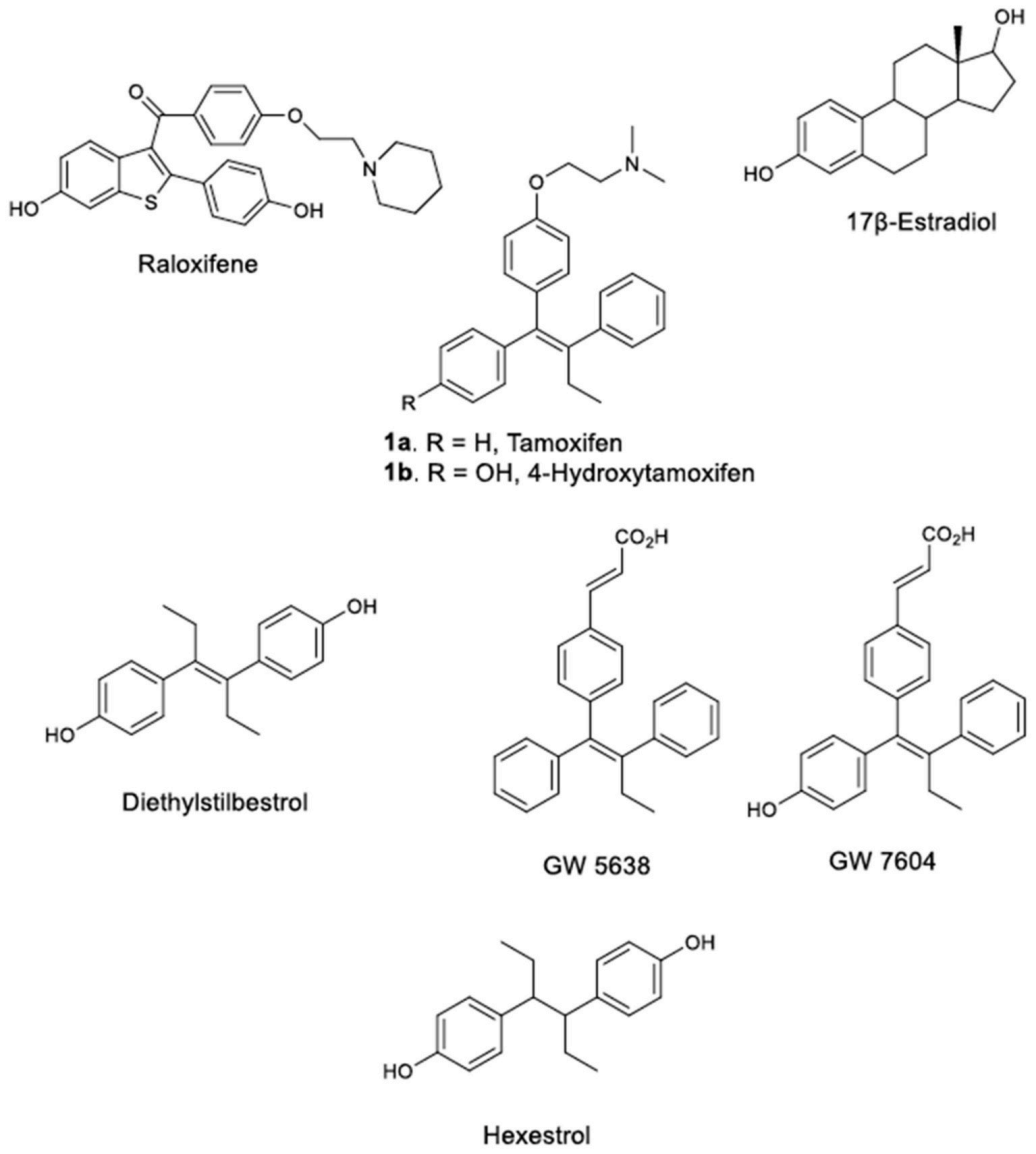
TAM, Estradiol and some of their analogs-derivatives.

The assumption of Lacassagne that the utilization of estrogen antagonists for the treatment of breast cancers developed by an inherited sensitivity to estrogens is capable of averting the progress of the disease has attracted considerable interest (Lacassagne, 1936; Jordan, 1999). Consequently, numerous estrogens antagonists (antiestrogens) have been synthesized and tested in respect to their binding affinity to the Estrogen Receptor (ER), a protein isolated and thoroughly studied (Toft and Gorski, 1966; Kuiper et al., 1996; Pettersson and Gustafsson, 2001). This campaign has afforded the discovery of various potent synthetic estrogens such as diethylstilbestrol (DES) (Lonning et al., 2001), and hexestrol (Figure 1), which have contributed greatly toward the development of a new philosophy for the design of antiestrogens (Dodds et al., 1938), which afforded the discovery of various novel stilbene derivatives with pronounced bioactivities, exemplified by the action of Tamoxifen (TAM) on hormone dependent breast cancers (Dellapasqua and Castiglione-Gertsch, 2005).

TAM (9-12), (Z)-2-[4-(1,2-diphenyl-1-butenyl)phenoxy]-N,N-dimethylethanamine (**1a**, Figure 1) is the endocrine treatment of choice for all stages of ER positive breast cancers and the first chemotherapeutic agent approved for the reduction of the breast cancers of high risk women. TAM displays therapeutic indexes and features that are clearly distinguishable from the other anticancer agents (Morello et al., 2002; Singh et al., 2008; Peng et al., 2009) and as its significant estrogen like properties are observed on specific target tissues it is considered one of the first Selective Estrogen Receptor Modulators (SERMs). SERMs act on specific target receptors by diffusing into the cell and binding to the Estrogen Receptors ERα and ERβ (Barkhem et al., 1998; Balfe et al., 2004; Jordan, 2004; Lewis and Jordan, 2005; Ahmed et al., 2016). After oral administration, TAM is extensively metabolized to (*Z*)-4-hydroxytamoxifen, (4OH-TAM, **1b**, Figure 1), a TAM metabolite exhibiting an 8-fold higher binding affinity to the ER as compared to TAM. On the other hand, 4OH-TAM is susceptible to isomerization and converted readily into a *Z*/*E* mixture, whereas the (*E*) isomer has only a 5% affinity to the ER (Robertson et al., 1982). Raloxifene (RAL), the second most popular SERM acts as an antiestrogen in breast and uterus and as estrogen in bone.

There are several literature reports on the X-ray crystallographic determination of the binding modes of diverse ligands such as estradiol, (EST), DES, 4OH-TAM, and RAL, in the Ligand Binding Domain (LBD) of ER isoforms ERα and ERβ (Brzozowski et al., 1997; Shiau et al., 1998). In this respect, the orientation of C-terminal Helix of ER LBD (Helix-12) is associated with the agonist, antagonist or partial agonist properties of a ligand and an equilibrium was proposed between the two-stable agonist-antagonist domains of Helix-12 (Steinmetz et al., 2001). A more recent study implies that the ER LBD displays structural plasticity, (PDB entry 2P15) when the ortho-trifluoromethylphenylvinyl estradiol (EZT), a bulky analog of estradiol, is bound inside the binding pocket forcing the Helix-12 to adopt the agonist conformation (Nettles et al., 2007).

Early TAM syntheses have faced a serious drawback concerning the prevailing stereochemistry obtained during the introduction of the alkane moiety. For this purpose, a considerable synthetic effort was devoted for the development of a stereospecific synthetic route for the desired isomer (Winkler et al., 1971; Miller and Al-Hassan, 1984; McCague, 1987; Detsi et al., 2002). Simultaneously, several methods were embraced for the separation of the isomers using either a selective crystallization procedure or various chromatographic techniques, such as the reversed phase high performance liquid chromatography (Manns et al., 1998) or the preparative thin layer chromatography. In most cases, the separation was performed at the final step, usually after the deprotection of the phenol protective groups, making the synthesis more convenient and applicable.

Although the use of long-term antiestrogen therapy to antagonize estrogen action in breast cancers has proven to be a very flourishing strategy for the treatment of ERα-positive breast cancer for post-menopausal women, it is also clear that the development of an improved antiestrogen therapy is essential to overcome the frequent problem of tumor resistance to hormonal therapy (Ring and Dowsett, 2004; Weinberg et al., 2005). Another serious drawback, not yet answered concerns the side effects of TAM in ovaries (Mourits et al., 1999; Metindir et al., 2005) and uterus (Ascher et al., 2000; Jones et al., 2012), since its antagonist character is only related with the breast tissue. In this respect, it is well established that substrates bearing the stilbene moiety display mixed antagonist-agonist or pure antagonist properties. On the contrary compounds containing the estradiol framework exhibit agonistic activity for both ERα and ERβ receptors (Hertrampf et al., 2008).

After the approval of TAM as medicine for the treatment of breast cancer, considerable research effort was initiated toward the discovery of novel TAM derivatives-analogs that will retain its antiestrogenic activity on breast tissues lacking its agonistic action on other tissues. Conclusively, receptor binding affinity is one of the key components in demonstrating antiestrogen activity. In this frame, the essential structural features necessary for high ligand binding affinity and antiestrogenic activity are extensively studied and reported and include: (1) The presence of an alkylaminoethoxy side chain, (2) the distance between the nitrogen and the oxygen should be optimal, (3) the conformation available to the side-chain should not be restricted, and (4) the nitrogen atom should demonstrate appropriate basicity.

Therefore, an appreciable number of novel compounds was synthesized and evaluated, most of them maintaining the ether functional group on their C-ring. Other compounds, such as triphenolic derivatives performed as high binding affinity antiestrogens (Lubczyk et al., 2003), whereas the presence of short alkyl- and polar amino-/amido-ethyl chains into TAM analogs (Kaur et al., 2016), inhibited the expression of specific proteins in both MCF-7 and MDA-MB-231 cells. On the other hand, there are only limited reports concerning the replacement of the ether moiety (Lubczyk et al., 2002), including the straightforward syntheses of numerous N-substituted TAM derivatives (Ahmed et al., 2016) containing a carbonyl group directly connected to the phenol C-ring. These compounds were determined to display selective estrogenic activities combined with good binding affinities to the ER (Rubin et al., 2001, 2002; Nguyen et al., 2007).

The principal aim of the presented work was to “reinvent” TAM and provide compounds with increased binding affinity for the Estrogen Receptors compared to landmark TAM and related molecules developed after its inauguration. Notwithstanding, future SERMs need to be utterly devoid of estrogenic activity to prevent the development of resistance, as it occurs with TAM. Considering that straightforward TAM derivatives maintaining the prerequisite of a nitrogen and oxygen atom with optimum distance between them, are vastly studied, a series of carbonyl side chain derivatives (see likewise previous work of our group; Christodoulou et al., 2013) were attached on TAM's ether functionality to prepare the corresponding ester derivatives and assess their biological properties. Furthermore, the benzoic acid group was introduced to construct several novel acid derivatives. The introduction of such moiety in TAM backbone resembles that of compound GW 7604 (Figure 1), which belongs to a class of compounds characterized as Selective Estrogen Receptor Downregulators (SERDs). These compounds, along with estradiol and ICI 182,780 (Faslodex), are well known to induce a rapid and sustained decrease in ERα protein (decrease cellular ERα levels).

Overall, the RBAs of the novel TAM derivatives were evaluated using purified preparations of ERα and ERβ for the determination of their ER binding affinities. Furthermore, a cell-based assay was used to assess their breast tissue activities. Finally, several theoretical calculations and molecular modeling were performed to investigate, support and explain the binding affinities and the biological profile of the new compounds.

## Materials and methods

### Chemistry

All anhydrous reactions were carried out under argon atmosphere. Solvents were dried by distillation prior to use. Solvent mixtures employed in chromatography were reported as volume to volume ratios. Starting materials were purchased from Aldrich (analytical reagent grades) and used without further purification. Analytical thin-layer chromatography (TLC) was conducted on Merck glass plates coated with silica gel 60 F_254_ and spots were visualized with UV light or/and an alcohol solution of anisaldehyde. Flash column chromatography was performed using Merck silica gel 60 (230–400 mesh ASTM).

Melting points were determined on a Büchi melting point apparatus and are uncorrected. ^1^H and 2D NMR spectra were recorded on a Bruker Advance III 600 MHz in the indicated solvents. The coupling constants are recorded in Hertz (Hz) and the chemical shifts are reported in parts per million (δ, ppm), downfield from tetramethylsilane (TMS) that was used as an internal standard (by asterisk are indicated the overlapped peaks).

Infrared spectra were obtained on a Nicolet Magna 750, series II spectrometer. HPLC separations were performed using an HP Agilent 1100 series instrument with a variable wavelength UV detector and coupled to HP Chem.–Station utilizing the manufacturer's 5.01 software package.

### 1,2-bis-(4-benzloxy-phenyl)-butan-1-one, 3

The synthesis of ethylated desoxyanisoin is described in a previously published procedure of our group (Kasiotis et al., 2001). Subsequently, deprotection of methoxy groups occurred under strong acidic conditions (Belanger et al., 1988), providing 1,2-bis-(4-hydroxyphenyl)-butan-1-one. After standard work up, and drying, the resulting product was used without further purification for the next step.

#### Procedure

To a solution of 1,2-bis-(4-hydroxyphenyl)-butan-1-one (0.2 g, 0.78 mmol) in DMF (2 mL) was added K_2_CO_3_ portionwise (0.2 g, 1.4 mmol) under continuous stirring. The resulting mixture was heated at 60°C and stirred for 4 h. Afterwards, the mixture was allowed to reach ambient temperature, BnBr (0.2 mL, 1.7 mmol) was added dropwise and stirring was prolonged for 12 h. The completion of the reaction was confirmed by TLC. Then a saturated solution of NH_4_Cl (5 mL) was added; the mixture was extracted with EtOAc (2 × 20 mL) and the combined organic layers were then washed with brine (5 mL) and water (5 mL) and dried with MgSO_4_. Evaporation under vacuum provided a yellow oily residue that was subjected to flash column chromatography (EtOAc/hexane 1:4) providing the desired compound **3**. White solid, yield 80%, mp 71–73°C. IR: $\tilde{\nu }=$ 1725 cm^−1^. ^1^H NMR (CDCl_3_): δ = 0.88 (t, *J* = 7.3Hz, 3H, -CH_2_CH_3_), 1.82 (m, 1H, -CH_2_CH_3_), 2.19 (m, 1H, -CH_2_CH_3_), 4.33 (t, *J* = 7.3 Hz, 1H, H-2), 5.01 (s, 2H, OCH_2_), 5.09 (s, 2H, OCH_2_), 6.91 (d, 2H, *J* = 8.8, ArH), 6.96 (d, *J* = 8.8 Hz, 2H, ArH), 7.28 (d, *J* = 8.8 Hz, 2H, ArH), 7.33–7.45 (m, 10H, ArH), 7.98 (d, *J* = 8.8 Hz, 2H,ArH). C_30_H_28_NO_3_ (436.54): calcd. C 82.54, H 6.46; found C 82.36, H 6.52.

##### Preparation of (E)-4-[1,2-bis-(4-benzyloxy-phenyl)-but-1-enyl]-phenol, 4a and (Z)-4-[1,2-bis-(4-benzyloxy-phenyl)-but-1-enyl]-phenol 4b

To a solution of (4-bromo-phenoxy)-tert-butyl-dimethyl-silane (0.35 g, 1.2 mmol) in THF (4 mL) was added n-butyllithium (1.6 M in hexane, 0.11 mL, 1.2 mmol) at −78°C. The resulting mixture was stirred for 10 min; then a solution of the ketone 3 (1.2 mmol) in THF (4 mL) was added, and stirring continued at −78°C for 1 h followed by 20 h at ambient temperature. Saturated solution of NH_4_Cl (1 mL) was added; the mixture was diluted with EtOAc (25 mL) and then washed with brine (5 mL) and water (5 mL). The organic layer was dried (MgSO_4_) and concentrated. The residues were dissolved in ethanol (8 mL) and HCl (37%, 2 mL). The mixture was heated to reflux for 2 h, allowed to cool, diluted with EtOAc (20 mL), and washed with water (10 mL), aqueous sodium thiosulfate solution (5 M, 5 mL), and water (15 mL). The organic layer was dried (MgSO_4_) and concentrated. Purification by flash chromatography (EtOAc/hexane 1:4) afforded the desired geometrical isomers 4a, 4b as 1:1 mixture (E/Z) (by 1H NMR). Separation by semi-preperative HPLC as mentioned resulted to the desired E-isomer. To both isomers, yield-50%, mp (referred to E-isomer) 78–79°C. IR: $\tilde{\nu }=$ 3,516, 3,264 cm^−1^. ^1^H NMR (CDCl_3_): δ = 0.89 (t, *J* = 7.1 Hz, 3H, -CH_2_CH_3_), 2.44 (q, *J* = 7.1 Hz, 2H, -CH_2_CH_3_), 5.01 (s, 2H, -OCH_2_-), 5.09 (s, 2H, -OCH_2_-), 6.55–7.41 (m, 22 H, ArH). C_36_H_32_O_3_ (512.64): calcd. C 84.35, H 6.29; found C 84.19, H 6.36.

[column: Kromasil 100-5, C-18, (25 × 10 mm); mobile phase CH_3_CN/H_2_O (4:1); detector: UV (λ = 300 nm); flow: 1.5 mL/min; load: 5 mg/100 μL of solution in mobile phase, t_R_4b = (7.1 min) and t_R_4a = (8.8 min)].

##### General procedure for the preparation of the halogen acetic acid phenyl esters 5a, 5b

The key intermediate mixture of **4a** and **4b** or only the diastereoisomer **4a** (0.93 mmol) was dissolved in ice-cold anhydrous diethylether (8 mL). Subsequently pyridine (0.186 mmol) and halogenacetylhalide (1.1 mmol) were added. The reaction was run under stirring at that temperature for 2 h, and then diluted with EtOAc (20 mL). The organic layer was separated, washed with water (20 mL) and dried over MgSO_4_. Purification by flash chromatography (EtOAc/hexane 1:4) afforded the desired compounds **5a**, **5b** as 11:1 mixture (by ^1^H NMR) of the Z/E diastereomers. Recrystallization from diethylether resulted in the separation of the pure Z isomers as white solid respectively.

##### Z-Chloro-acetic acid 4-[1,2-bis-(4-benzyloxy-phenyl)-but-1-enyl]-phenyl ester, 5a

White solid, yield 90%, mp 92–94°C. IR: $\tilde{\nu }=$ 1761 cm^−1^. ^1^H NMR (CDCl_3_): δ = 0.89 (t, *J* = 7.1 Hz, 3H, -CH_2_CH_3_), 2.44 (q, *J* = 7.2 Hz, 2H, -CH_2_CH_3_), 4.08 (s, 2H, CH_2_-Cl), 5.01 (s, 2H, OCH_2_), 5.10 (s, 2H, OCH_2_), 6.61–7.01 (m, 22H, ArH). C_38_H_33_ClO_4_ (589.12): calcd. C 77.47, H 5.65, Cl.6.02; found C 77.36, H 5.70, Cl.5.90.

##### Z-Bromo-acetic acid 4-[1,2-bis-(4-benzyloxy-phenyl)-but-1-enyl]-phenyl ester, 5b

Yellowish solid, yield 90%, mp 95–96°C. IR: $\tilde{\nu }=$ 1759 cm^−1^. ^1^H NMR (CDCl_3_): δ = 0.90 (t, *J* = 7.1 Hz, 3H, -CH_2_CH_3_), 2.45 (q, *J* = 7.2 Hz, 2H, -CH_2_CH_3_), 4.05 (s, 2H, CH_2_-Br), 4.99 (s, 2H, OCH_2_), 5.08 (s, 2H, OCH_2_), 6.58–7.52 (m, 22H, ArH). C_38_H_33_BrO_4_ (633.57): calcd. C 72.04, H 5.25, Br 12.61; found C 72.20, H 5.22, Br 12.51.

##### General procedure for the preparation of the dimethylamino acetic acid phenyl ester 7a diethylamino acetic acid phenyl ester 7b and morpholin-4-yl acetic acid phenyl ester 7c

In a solution of **5a** or **5b** or **5c** (0.23 mmol) in 4 mL of THF was added triethylamine (1.65 mmol) and the appropriate amine (11.80 mmol). The resulting mixture was heated at 40°C for 1 h and allowed to reach room temperature. Then the reaction was quenched with a saturated solution of NH_4_Cl (20 mL) and subsequently extracted with EtOAc (2 × 20 mL). The combined organic layers were washed with brine, dried over MgSO_4_ and concentrated under reduced pressure. Purification by flash chromatography (EtOAc/hexane 3:7) afforded the desired compounds **7a**, **7b**, and **7c** as pure Z-diastereomers.

##### Z-Dimethylamino-acetic acid 4-[1,2-bis-(4-benzyloxy-phenyl)-but-1-enyl]-phenyl ester (7a)

White solid, yield 41%, mp 80–82°C. IR: $\tilde{\nu }=$ 1,749 cm−1. ^1^H NMR (CDCl_3_): δ = 0.92 (t, *J* = 7 Hz, 3H, -CH_2_CH_3_), 2.35–2.53 (m, 8H, -CH_2_CH_3_, -NCH_3_), 3.44 (s, 2H, -COCH_2_-), 4.98 (s, 2H, OCH_2_), 5.06 (s, 2H, OCH_2_), 6.62–7.39 (m, 22H, ArH). C_40_H_39_NO_4_ (597.74): calcd. C 80.37, H 6.58, N 2.34; found C 80.24, H 6.62, N 2.39.

##### Z-Diethylamino-acetic acid 4-[1,2-bis-(4-benzyloxy-phenyl)-but-1-enyl]-phenyl ester 7b

White solid, yield 60%, mp 79–80°C. IR: $\tilde{\nu }=$ 1,755 cm^−1^. ^1^H NMR (CDCl_3_): δ = 0.96 (t, *J* = 7 Hz, 3H, -CH_2_CH_3_), 1.12 (t, *J* = 7 Hz, 6H, -NCH_2_CH_3_), 2.51 (q, *J* = 7 Hz, 2H, -CH_2_CH_3_), 2.75 (q, *J* = 7 Hz, 4H, -NCH_2_CH_3_), 3.55 (s, 2H, -COCH_2_), 5.04 (s, 2H, OCH_2_), 5.12 (s, 2H, OCH_2_), 6.72–7.54 (m, 22H, ArH). C_42_H_43_NO_4_ (625.8): calcd. C 80.61, H 6.93, N 2.24; found C 80.75, H 6.83, N 2.30.

##### Z-Morpholin-4-yl-acetic acid 4-[1,2-bis-(4-benzyloxy-phenyl)-but-1-enyl]-phenyl ester (7c)

White solid, yield 65%, mp 71–73°C. IR: $\tilde{\nu }=$ 1,761 cm^−1^. ^1^H NMR (CDCl_3_): δ = 0.96 (t, *J* = 7 Hz, 3H, -CH_2_CH_3_), 2.44 (q, *J* = 7 Hz, 2H, -CH_2_CH_3_), 2.62 (m, 4H, -NCH_2_CH_2_), 3.51 (s, 2H, -COCH_2_-), 3.62 (m, 4H, CH_2_CH_2_O-), 5.04 (s, 2H, CH_2_O), 5.11 (s, 2H, CH_2_O), 6.77–7.52 (m, 22H, ArH). C_42_H_42_NO_5_ (639.78): calcd. C 78.85, H 6.46, N 2.19; found C 78.69, H 6.62, N 2.29.

##### General procedure for the preparation of halogen acetates 6a, 6b, dimethylamino acetate 8a, diethylamino acetate 8b, morpholin-4-yl acetate 8c

Alkenes **5a, 5b, 7a, 7b, 7c** were dissolved in ethyl acetate and hydrogenated over 10% Pd/C under 1 atmosphere pressure for 4 h in the absence of sunlight. The mixtures were filtered over Celite, dried over MgSO_4_ and evaporated. Purification by flash chromatography (EtOAc/hexane 1:1) afforded the desired compounds.

##### Z-Chloro-acetic acid 4-[1,2 bis-(4-hydroxy-phenyl)-but-1-enyl]-phenyl ester (6a)

White solid, yield 75%, mp 131–133°C. IR: $\tilde{\nu }=$ 3,523, 1745 cm^−1^. ^1^H NMR ([D6]acetone): δ = 0.96 (t, *J* = 7.3 Hz, 3H, -CH_2_CH_3_), 2.44 (q, *J* = 7.3 Hz, 2H, -CH_2_CH_3_), 4.29 (s, 2H, CH_2_-Cl), 6.63 (d, *J* = 8.6 Hz, 2H, ArH), 6.73 (d, *J* = 8.3 Hz, 2H, ArH), 6.79 (d, *J* = 8.6 Hz, 2H, ArH), 7.00 (d, *J* = 8.3 Hz, 2H, ArH), 7.21 (d, *J* = 8.6 Hz, 2H, ArH), 7.35 (d, *J* = 8.6 Hz, 2H, ArH), 8.25 (bs, 1H, OH), 8.29 (bs, 1H, OH). C_24_H_21_ClO_4_ (408.87): calcd. C 70.50, H 5.18, N 8.67; found C 70.67, H 5.23, N 8.49.

##### Z-Bromo-acetic acid 4-[1,2 bis-(4-hydroxy-phenyl)-but-1-enyl]-phenyl ester (6b)

White solid, yield 80%, mp 133–135°C. IR: $\tilde{\nu }=$ 3,523, 1745 cm^−1^. ^1^H NMR ([D6]acetone): δ = 0.96 (t, *J* = 7.3 Hz, 3H, -CH_2_CH_3_), 2.44 (q, *J* = 7.3 Hz, 2H, -CH_2_CH_3_), 4.35 (s, 2H, CH_2_-Br), 6.58 (d, *J* = 8.6 Hz, 2H, ArH), 6.70 (d, *J* = 8.3 Hz, 2H, ArH), 6.75 (d, *J* = 8.6 Hz, 2H, ArH), 6.99 (d, *J* = 8.3 Hz, 2H, ArH), 7.21 (d, *J* = 8.3 Hz, 2H, ArH), 7.31 (d, *J* = 8.6 Hz, 2H, ArH), 8.22 (bs, 1H, OH), 8.32 (bs, 1H, OH). C_24_H_21_BrO_4_ (453.33): calcd. C 63.59, H 4.67, N 17.63; found C 63.46, H 4.62, N 17.79.

##### Z-Dimethylamino-acetic acid 4-[1,2-bis-(4-hydroxy-phenyl)-but-1-enyl]-phenyl ester (8a)

Pale yellowish solid, yield 72%. IR: $\tilde{\nu }=$ 3,518, 3,264, 1,760 cm^−1^. ^1^H NMR ([D_6_]acetone): δ = 0.92 (t, *J* = 7 Hz, 3H, -CH_2_CH_3_), 2.35–2.53 (m, 8H, -CH_2_CH_3_, -NCH_3_), 3.47 (s, 2H, -COCH_2_-), 6.58–7.25 (m, 12H, ArH). C_26_H_27_NO_4_ (330.4): calcd. C 74.80, H 6.52, N 3.35; found C 74.96, H 6.61, N 3.27.

##### Z-Diethylamino-acetic acid 4-[1,2-bis-(4-hydroxy-phenyl)-but-1-enyl]-phenyl ester (8b)

Pale yellowish oil, yield 50%°. IR: $\tilde{\nu }=$ 3,523, 3,264, 1,755 cm^−1^. ^1^H NMR ([D6]acetone): δ = 0.91 (t, *J* = 7 Hz, 3H, -CH_2_CH_3_), 1.07 (m, 6H, -NCH_2_CH_3_), 2.45 (q, *J* = 7 Hz, 2H, -CH_2_CH_3_), 2.71 (m, 4H, -NCH_2_CH_3_), 3.55 (s, 2H, -COCH_2_), 6.51–7.32 (m, 12H, ArH). C_28_H_31_NO_4_ (445.55): calcd. C 75.48, H 7.01, N 3.14; found C 75.36, H 6.94, N 3.21.

##### Z-Morpholin-4-yl-acetic acid 4-[1,2-bis-(4-hydroxy-phenyl)-but-1-enyl]-phenyl ester (8c)

White solid, yield 75%, mp 142–143°C. IR: $\tilde{\nu }=$ 3,521, 1,745 cm^−1^. ^1^H NMR ([D6]acetone): δ = 0.96 (t, *J* = 7 Hz, 3H, -CH_2_CH_3_), 2.43 (q, *J* = 7 Hz, 2H, -CH_2_CH_3_), 2.61 (m, 4H, -NCH_2_CH_2_O), 3.51 (s, 2H, -COCH_2_), 3.61 (m, 4H, -NCH_2_CH_2_O), 6.93–7.41 (m, 12H, ArH). C_28_H_29_NO_5_ (459.53): calcd. C 73.18, H 6.36, N 3.05; found C 73.33, H 6.44, N 3.10.

##### (E)-4-[1,2-bis-(4-benzyloxy-phenyl)-but-1-enyl]-benzoic acid, 9E

##### (Z)-4-[1,2-bis-(4-benzyloxy-phenyl)-but-1-enyl]-benzoic acid, 9Z

To a solution of 4-bromo-benzoic acid (0.65 g, 1.49 mmol) in THF (8 mL) was added *n*-butyllithium (1.6 M in hexane, 3.98 mL, 2.98 mmol) at −78°C. The resulting mixture was stirred for 10 min; then a solution of ketone **3** (1.4 g, 1.49 mmol) in THF (7 mL) was added, and stirring continued at −78°C for 1 h followed by 20 h at ambient temperature. Saturated solution of NH_4_Cl (1 mL) was added; the mixture was diluted with EtOAc (25 mL) and then washed with brine (5 mL) and water (5 mL). The organic layer was dried (MgSO_4_) and concentrated. The residues were dissolved in ethanol (8 mL) and HCl (37%, 2 mL). The mixture was heated to reflux for 2 h, allowed to cool, diluted with EtOAc (20 mL), and washed with water (10 mL), aqueous sodium thiosulfate solution (5 M, 5 mL), and water (15 mL). The organic layer was dried (MgSO_4_) and concentrated. Purification by flash chromatography (EtOAc/hexane 2:3) afforded the desired geometrical isomers **9E, 9Z** as 5:1 mixture respectively. Separation by semi-preperative HPLC furnished, as mentioned, the pure isomers (Yield referred to both isomers, 0.29 g, 37%). M.p. (E*-*isomer) 195–197°C. IR: 3516, 3264, 1682 cm ^−1^.

**9E:** ^1^H NMR (DMSO-d6): δ = 0.83 (t, *J* = 7.1 Hz, 3H, -CH_2_CH_3_), 2.39 (q, *J* = 7.1 Hz, 2H, -CH_2_CH_3_), 4.91 (s, 2H, -OCH_2_-), 4.99 (s, 2H, -OCH_2_-), 6.70 (m, 1H, ArH), 6.81 (d, *J* = 8.3 Hz, 2H, ArH), 6.91 (d, *J* = 8.1 Hz, 2H, ArH), 6.99 (d, *J* = 8.8 Hz, 4H, ArH), 7.09 (d, *J* = 8.6 Hz, 2H, ArH), 7.24–7.39 (m, 7H, ArH), 7.43 (d, *J* = 8.6 Hz, 2H, ArH), 7.89 (d, *J* = 8.1 Hz, 2H, ArH). Anal. Calcd forC_37_H_32_O_4_: C 82.20, H 5.97. Found: C 82.43, H 6.07.

**9Z:** ^1^H NMR (DMSO-d6): δ = 0.83 (t, *J* = 7.1 Hz, 3H, -CH_2_CH_3_), 2.39 (q, *J* = 7.1 Hz, 2H, -CH_2_CH_3_), 4.97 (s, 2H, -OCH_2_-), 5.06 (s, 2H, -OCH_2_-), 6.70 (m, 1H, ArH), 6.79 (d, *J* = 8.3 Hz, 2H, ArH), 6.91 (d, *J* = 8.1 Hz, 2H, ArH), 6.99 (d, *J* = 8.8 Hz, 4H, ArH), 7.09 (d, *J* = 8.6 Hz, 2H, ArH), 7.24–7.39 (m, 7H, ArH), 7.43 (d, *J* = 8.6 Hz, 2H, ArH), 7.58 (d, *J* = 8.1 Hz, 2H, ArH). Anal. Calcd forC_37_H_32_O_4_: C 82.20, H 5.97. Found: C 81.93, H 6.04.

[column: Kromasil 100-5, C-18, (25 × 10 mm); mobile phase CH_3_CN/H_2_O (3:1); detector: UV (λ = 300 nm); flow: 1.5 mL/min; load: 5 mg/100 μL of solution in mobile phase, t_R_**9Z** = (5.3 min) and t_R_**9E** = (7.2 min)].

##### (E)-4-[1,2-bis-(4-benzyloxy-phenyl)-but-1-enyl]-n-(2-bromo-ethyl)-benzamide, 11E

##### (Z)-4-[1,2-bis-(4-benzyloxy-phenyl)-but-1-enyl]-n-(2-bromo-ethyl)-benzamide, 11Z

To a solution of ketone **9Z, 9E** (0.10 g, 0.18 mmol) in anhydrous THF (3.0 mL) was added dropwise thionylchloride (0.06 mL, 0.82 mmol). Subsequently the solution was heated at 60°C, stirred for 30 min, allowed to cool and neutralized with a saturated solution of NaHCO_3_ (3 mL). The mixture was diluted with EtOAc (25 mL), washed with water (5 mL) and the organic layer was dried (MgSO_4_) and concentrated. In the resulting residue (dissolved in anhydrous DMF, 2 mL) was added slowly a solution of 2-bromoethylamine hydrobromide (82 mg, 0.40 mmol) and triethylamine (0.03 mL, 0.21 mmol) in anhydrous DMF (2 mL). The mixture was heated to 100°C for 1 h, allowed to reach room temperature, neutralized with NH_4_Cl (2 mL) and diluted with water (12 mL). The product was extracted with two portions of EtOAc (15 mL), washed with water, brine and dried over MgSO_4_. Purification by flash chromatography (EtOAc/hexane 1:4) afforded the desired product (60 mg, 50% yield) as a mixture of the two isomers **11E/11Z (**7:1). Crystallization from diethyl ether resulted in the separation of the pure E isomer as pale white solid.

Analytically pure samples of the latter compounds were obtained by semi–preparative HPLC [column: Kromasil 100-5, C-18, (25 × 10 mm); mobile phase CH_3_CN/H_2_O/trichloro acetic acid (3:1.8:0.1); detector: UV (λ = 300 nm); flow: 1.6 mL/min; load: 5 mg/100 μL of solution in mobile phase, t_R_**11Z** = (6.1 min) and t_R_**11E** = (7.8 min)].

**11E***:* White solid. M.p. 152–153°C. IR: 1701 cm ^−1^. ^1^H NMR (CDCl_3_): δ = 0.92 (t, *J* = 7.3 Hz, 3H, -CH_2_C*H*_3_), 2.47 (q, *J* = 8.3 Hz, 2H, -C*H*_2_CH_3_), 4.01 (t, *J* = 9.4 Hz, 2H, - NHCH_2_C*H*_2_Br), 4.37 (t, *J* = 9.4 Hz, 2H, -NHC*H*_2_CH_2_Br), 4.93 (s, 2H, -OC*H*_2_-), 5.01 (s, 2H, -OC*H*_2_-), 6.65 (d, 2H, *J* = 9.1 Hz, ArH), 6.76 (d, 2H, *J* = 8.8 Hz, ArH), 6.80 (d, *J* = 8.8 Hz, 2H, ArH), 7.13 (d, *J* = 8.8 Hz, 2H, ArH), 7.25–7.45 (m, 12H, ArH), 8.06 (d, *J* = 8.5 Hz, 2H, ArH). Anal. Calcd for C_39_H_36_BrNO_3_: C 72.44, H 5.61, Br 12.36, N 2.17. Found: C 72.63, H 5.71, Br 12.24, N 2.09.

**11Z***:* ^1^H NMR (CDCl_3_): δ = 0.92 (t, *J* = 7.3 Hz, 3H, -CH_2_CH_3_), 2.41 (q, *J* = 8.3 Hz, 2H, -CH_2_CH_3_), 4.08 (t, *J* = 9.4 Hz, 2H, -CH_2_Br -), 4.43 (t, *J* = 9.4 Hz, 2H, -NHCH_2_-), 4.99 (s, 2H, -OCH_2_-), 5.08 (s, 2H, -OCH_2_-), 6.68 (d, 2H, *J* = 9.1 Hz, ArH), 6.76 (d, 2H, *J* = 8.8 Hz, ArH), 6.80 (d, *J* = 8.8 Hz, 2H, ArH), 7.06 (d, *J* = 8.8 Hz, 2H, ArH), 7.25–7.45 (m, 12H, ArH), 7.92 (d, *J* = 8.5 Hz, 2H, ArH). Anal. Calcd for C_39_H_36_BrNO_3_: C 72.44, H 5.61, Br 12.36, N 2.17. Found: C 72.68, H 5.74, Br 12.14, N 2.28.

##### (E)-4-[1,2-bis-(4-benzyloxy-phenyl)-but-1-enyl]-n-(3-bromo-propyl)-benzamide, 12E

##### (Z)-4-[1,2-bis-(4-benzyloxy-phenyl)-but-1-enyl]-n-(3-bromo-propyl)-benzamide, 12Z

To a solution of ketone **9Z, 9E** (0.12 g, 0.21 mmol) in anhydrous THF (3.0 mL) was added dropwise thionylchloride (0.06 mL, 0.82 mmol). Subsequently the solution was heated at 60 °C, stirred for 30 min, allowed to cool and neutralized with a saturated solution of NaHCO_3_ (3 mL). The mixture was diluted with EtOAc (25 mL), washed with water (5 mL) and the organic layer was dried (MgSO_4_) and concentrated. In the resulting residue (dissolved in anhydrous DMF, 2 mL) was added slowly a solution of 3-bromopropylamine hydrobromide (0.12 g, 0.40 mmol) and triethylamine (0.03 mL, 0.21 mmol) in anhydrous DMF (2 mL). The mixture was heated to 100°C for 1 h, allowed to reach room temperature, neutralized with NH_4_Cl (2 mL) and diluted with water (15 mL). The product was extracted with two portions of EtOAc (15 mL), washed with water, brine and dried over MgSO_4_. Purification by flash chromatography (EtOAc/hexane 1:4) afforded the desired product (72 mg, 50% yield) as a mixture of the two isomers **12E/12Z** (10:1). Repetitive recrystallization from methylene chloride-petroleum ether resulted in the separation of the pure E isomer as white solid. Analytically pure samples of the latter compounds were obtained by semi–preparative HPLC [column: Kromasil 100-5, C-18, (25 × 10 mm); mobile phase CH_3_CN/H_2_O/trichloro acetic acid (3.4:1.9:0.1); detector: UV (λ = 300 nm); flow: 1.6 mL/min; load: 5 mg/100 μL of solution in mobile phase, t_R_**12Z** = (5.9 min) and t_R_**12E** = (7.9 min)].

**12E**: White solid, M.p. 164–166°C. IR: 1714 cm^−1^. ^1^H NMR (CDCl_3_): δ = 0.90 (t, *J* = 7.3 Hz, 3H, -CH_2_CH_3_), 2.21 (m, 2H, -NHCH_2_CH_2_CH_2_Br), 2.39 (q, *J* = 7.3 Hz, 2H, -CH_2_CH_3_), 3.51 (t, *J* = 6.2 Hz, 2H, -NHCH_2_CH_2_CH_2_Br), 3.61 (t, *J* = 6.2 Hz, 2H, -NCH_2_CH_2_CH_2_Br), 4.91 (s, 2H, -OCH_2_), 5.01 (s, 2H, -OCH_2_-), 6.34 (t, *J* = 6.2 Hz, 1H, -NH), 6.64 (d, *J* = 8.8 Hz, 2H, ArH), 6.74 (d, *J* = 8.8 Hz, 2H, ArH), 6.79 (d, *J* = 8.8 Hz, 2H, ArH), 6.94 (m, 1H, ArH), 7.12 (d, *J* = 8.8 Hz, 2H, ArH), 7.24–7.44 (m, 11H, ArH), 7.72 (d, *J* = 8.1 Hz, 2H, ArH). Anal. Calcd for C_40_H_38_BrNO_3_: C 72.72, H 5.80, Br 12.09, N 2.12. Found: C 72.93, H 5.71, Br 12.14, N 2.06.

**12Z***:* Pale yellow solid. M.p. 169–171°C. IR: 1704 cm^−1^. ^1^H NMR (CDCl_3_): δ = 0.90 (t, *J* = 7.3 Hz, 3H, -CH_2_CH_3_), 2.11 (m, 2H, -NHCH_2_CH_2_CH_2_Br), 2.43 (q, *J* = 7.3 Hz, 2H, -CH_2_CH_3_), 3.45 (t, *J* = 6.2 Hz, 2H, -NHCH_2_CH_2_CH_2_Br), 3.61 (t, *J* = 6.2 Hz, 2H, -NCH_2_CH_2_CH_2_Br), 4.97 (s, 2H, -OCH_2_-), 5.05 (s, 2H, -OCH_2_-), 6.19 (t, *J* = 6.2 Hz, 1H, -NH), 6.64 (d, *J* = 8.8 Hz, 2H, ArH), 6.74 (d, *J* = 8.8 Hz, 2H, ArH), 6.79 (d, *J* = 8.8 Hz, 2H, ArH), 6.94 (m, 1H, ArH), 7.03 (d, *J* = 8.8 Hz, 2H, ArH), 7.24–7.44 (m, 11H, ArH), 7.72 (d, *J* = 8.1 Hz, 2H, ArH). Anal. Calcd for C_40_H_38_BrNO_3_: C 72.72; H 5.80; Br 12.09; N 2.12. Found: C 72.86; H 5.74; Br 12.01; N 2.19.

#### General procedure for debenzylation

The mixture of **Z, E** isomers (**9**,**11,12**) was dissolved in EtOAc/or MeOH and hydrogenated over 10% Pd/C under 1 atmosphere pressure for 1–13 h in the absence of sunlight. The mixtures were filtered over Celite, dried over MgSO_4_ and evaporated. Purification by flash chromatography afforded the desired compounds.

##### (E)-4-[1,2-bis-(4-hydroxy-phenyl)-but-1-enyl]-benzoic acid, 10E

##### (Z)-4-[1,2-bis-(4-hydroxy-phenyl)-but-1-enyl]-benzoic acid, 10Z

Compound **9** was dissolved in MeOH and debenzylated according to the general procedure. Purification by flash chromatography (EtOAc/hexane 1:1) afforded the desired mixture of isomers. Analytically pure samples of the latter compounds were obtained by semi–preparative HPLC [column: Kromasil 100-5, C-18, (25 × 10 mm); mobile phase CH_3_CN/H_2_O/trichloro acetic acid (2:3:0.1); detector: UV (λ = 300 nm); flow: 1.6 mL/min; load: 5 mg/100 μL of solution in mobile phase, t_R_**10Z** = (2.8 min) and t_R_**10E** = (4.5 min)].

**10E:** White solid. M.p. 217–218°C. IR: 3523, 3259, 1685 cm^−1^. ^1^H NMR (DMSO-d6): δ = 0.83 (t, *J* = 7.1 Hz, 3H, -CH_2_CH_3_), 2.29 (q, *J* = 7.1 Hz, 2H, -CH_2_CH_3_), 6.42 (d, *J* = 8.3 Hz, 2H, ArH), 6.58 (d, *J* = 8.3 Hz, 2H, ArH), 6.60 (d, *J* = 8.7 Hz, 1H, ArH), 6.88 (d, *J* = 8.3 Hz, 2H, ArH), 7.25 (d, *J* = 7.9 Hz, 2H, ArH), 7.31–7.42 (m, 1H, ArH), 7.91 (d, *J* = 7.9 Hz, 2H, ArH), 9.2 (br s, 2H, -O*H*). Anal. Calcd for C_23_H_20_O_4_: C 76.65, H 5.59. Found: C 76.43, H 5.40.

**10Z**: White solid. M.p. 209–210°C. IR: 3523, 3259, 1685 cm ^−1^. ^1^H NMR (DMSO-d6): δ = 0.87 (t, *J* = 7.1 Hz, 3H, -CH_2_CH_3_), 2.32 (q, *J* = 7.1 Hz, 2H, -CH_2_CH_3_), 6.38 (d, *J* = 8.3 Hz, 2H, ArH), 6.59 (d, *J* = 8.3 Hz, 2H, ArH), 6.65 (d, *J* = 8.7 Hz, 1H, ArH), 6.91 (d, *J* = 8.3 Hz, 2H, ArH), 7.23 (d, *J* = 7.9 Hz, 2H, ArH), 7.31–7.38 (m, 1H, ArH), 7.91 (d, *J* = 7.9 Hz, 2H, ArH), 9.10 (br s, 2H, -OH). Anal. Calcd for C_23_H_20_O_4_: C 76.65, H 5.59. Found: C 76.48, H 5.69.

[column: Kromasil 100-5, C-18, (25 × 10 mm); mobile phase CH_3_CN/H_2_O/trichloro acetic acid (3:2:0.1); detector: UV (λ = 300 nm); flow: 1.6 mL/min; load: 5 mg/100 μL of solution in mobile phase, t_R_**10Z** = (3.7 min) and t_R_**10E** = (5.5 min)].

##### (E)-4-[1,2-bis-(4-hydroxy-phenyl)-but-1-enyl]-n-(2-bromo-ethyl)-benzamide, 13

A stirred EtOAc solution of **11E** (40 mg, 0.06 mmol) was hydrogenated for 22 h according to the general debenzylation procedure. The crude mixture was purified by flash chromatography (EtOAc/hexane, 3:2) to give 18 mg of diphenol **13** as a pale yellow solid (52%). M.p. 171–172°C. IR: 3411, 3314, 1701 cm^−1^. ^1^H NMR (DMSO-d6): δ = 0.92 (t, *J* = 7.3 Hz, 3H, -CH_2_CH_3_), 2.41 (q, *J* = 7.3 Hz, 2H, -CH_2_CH_3_), 4.05 (t, *J* = 9.8 Hz, 2H, -NHCH_2_CH_2_Br), 4.23 (t, *J* = 9.8 Hz, 2H, -NHCH_2_CH_2_Br), 6.53 (d, 2H, *J* = 8.8 Hz, ArH), 6.61–6.71 (m, 2H, ArH), 6.82 (d, *J* = 8.3 Hz, 1H, ArH), 6.98–7.02 (m, 1H, ArH), 7.05–7.10 (m, 2H, ArH), 7.32 (d, *J* = 8.3 Hz, 2H, ArH), 7.59 (d, *J* = 8.3 Hz, 1H, ArH), 7.94 (d, *J* = 8.3 Hz, 1H, ArH). Anal. Calcd for C_25_H_24_BrNO_3_: C 64.38, H 5.19, Br 17.13, N 3.00. Found: C 64.12, H 5.29, Br 17.30, N 3.11.

##### (E)-4-[1,2-bis-(4-hydroxy-phenyl)-but-1-enyl]-n-(3-bromo-propyl)-benzamide, 14

A stirred MeOH solution of **12** (0.13 g, 0.20 mmol) was hydrogenated for 1 h according to the general debenzylation procedure. The crude mixture was purified by flash chromatography (EtOAc/hexane, 1:1) to give 73 mg of phenol **14** as a pale yellow solid (80%). M.p. 127–129°C. IR: 3,501, 3,309, 1,685 cm^−1^. ^1^H NMR (DMSO-d6): δ: 0.78 (t, *J* = 7.3 Hz, 3H, -CH_2_CH_3_), 2.12 (m, 4H, -NHCH_2_CH_2_CH_2_Br, -CH_2_CH_3_), 3.55 (m, 4H, -NHCH_2_CH_2_CH_2_Br, -NHCH_2_CH_2_CH_2_Br), 6.51 (d, *J* = 8.8 Hz, 1H, ArH), 6.62 (d, *J* = 8.8 Hz, 1H, ArH), 6.82 (m, 1H, ArH), 6.95 (d, *J* = 8.8 Hz, 1H, ArH), 7.05 (d, *J* = 8.3 Hz, 1H, ArH), 7.12 (d, *J* = 8.3 Hz, 1H, ArH), 7.61 (d, *J* = 8.3 Hz, 1H, ArH), 7.73 (m, 1H, ArH), 7.87 (d, *J* = 8.3 Hz, 2H, ArH), 8.01 (d, *J* = 8.3 Hz, 1H, ArH), 8.15 (m, 1H, ArH). Anal. Calcd for C_26_H_26_BrNO_3_: C 65.00, H 5.46, Br 16.63, N 2.92. Found: C 65.21, H 5.54, Br 16.44, N 3.01. Indicative NMR of compounds are provided in Supplementary Material.

### Cell culture and assessment of cell proliferation

Cell proliferation was assessed using MCF-7 human mammary adenocarcinoma cells, as already described (Fokialakis et al., 2004). Briefly, cells that have been cultured and subcultured as recommended by the supplier (ATCC), were plated in 96-flat-bottomed-well microplates at a density of 10,000 cells/well in Dulbecco's MEM devoid of phenol-red and supplemented with 10% dextran coated charcoal (DCC)-treated fetal bovine serum (FBS). Serial dilutions of the test compounds were added to the cells 24 h after plating, and after incubation for 6 days with both medium and test compounds being renewed every 48 h, the number of viable cells was determined using the conventional conversion of MTT [3-(4,5-dimethylthiazol-2-yl)-2,5-diphenyltetrazolium bromide] (Sigma St Louis, MO, USA) to colored formazan. Cells that received 0.1 nM E2 (Sigma St Louis, MO, USA) served as stimulated proliferation controls, whereas those that received vehicle (DMSO to a final concentration ≤ 0.2%) only, served as basal proliferation controls. The pure antiestrogen ICI 182,780 (Tocris Bioscience, Ellisville, Missouri, USA) was used to inhibit the estrogenic response as stated in the text. Cells were also treated in serum-free medium for the indicated times with 4-OH TAM (Tocris Bioscience, Ellisville, Missouri, USA).

### Binding to isolated human ERα and ERβ

The binding affinities of the TAM derivatives relative to that of estradiol (relative binding affinity, RBA) for isolated ERα and ERβ (RBAα and RBAβ) were assessed using a Beacon 2000 Fluorescence Polarization Reader (Invitrogen) as previously described (Fokialakis et al., 2004). Briefly, we determined the concentrations of estradiol, **6a,b, 8a-c**, **10, 13**, and **14**, that inhibited the binding of the fluorescent estrogen ES2 (Invitrogen) to the isolated recombinant human ERα or ERβ (Invitrogen) by 50% (IC_50_), and used them to derive the RBA values of Table 1 as described in the legend to the Table.

**Table 1:** RBA values of TAM analogs.

**Product code**	**RBAα^a^**	**RBAβ^a^**
**6a**	120.08 ± 8.5	62.14 ± 4.3
**6b**	96.37 ± 7.2	82.39 ± 4.9
**8a**	101.35 ± 10.2	72.72 ± 2.1
**8b**	108.12 ± 11.9	77.37 ± 4.5
**8c**	8.06 ± 0.9	16.79 ± 1.4
**10**	12.2 ± 2.3	16.3 ± 3.9
**13**	17.4 ± 3.0	8.40 ± 2.5
**14**	7.94 ± 0.6	6.15 ± 0.6
Estradiol	100	100
4-OH TAM	40.04	22.2

a *The RBA values (mean ± SEM of at least three independent experiments) for ERα (RBAα) and ERβ (RBAβ) were calculated by [(IC_50_ estradiol/IC_50_ derivative) × 100], where IC_50_ values are estradiol and derivative concentrations capable of inhibiting binding of the fluorescent estrogen ES2 (1 nM) to ERα and ERβ by 50%. IC_50_ values of estradiol for ERα and ERβ were 3.42 ± 0.99 and 2.87 ± 0.64, respectively. The RBAα and RBAβ of estradiol were set equal to 100*.

### Theoretical calculations

#### Software

The Maestro software (Schrödinger Inc.) was used for structure preparation and visualization. All rigid docking calculations were carried out with Glide 3.5 Software (Schrödinger Inc.) and all flexible docking calculations were carried out with Macromodel Software 9.0 (Schrödinger Inc.). Partial charges for all ligands were calculated using Jaguar 4.2 software (Schrödinger Inc.).

#### Receptor preparation

PDB entry 3ERD and 2P15 was used as starting structure for LBD-ERα having H12 in agonist position, in complex with DES and EZT respectively, while PDB entry 3ERT was used as a starting structure for LBD-ERα having H12 in antagonist position, in complex with 4OH-TAM. The crystallographic ligand was removed; all crystallographic water molecules were deleted except the one between ARG 394 and GLU 353. HIS 524 was protonated on NE2 and the protein preparation module were used as implemented on Glide 3.5.

#### Ligand preparation

All ligands were designed using Maestro software. Partial charges were calculated using Jaguar 4.2 software (Schrödinger Inc.). 500 steps of Monte Carlo/Low Mode (MC-LMOD) search were run for each ligand with OPLS2003 force field and the global minimum structure were used as starting structure for docking calculations.

#### Docking calculations

Two different docking algorithms were used. First, rigid docking calculations were run (Glide 3.5) where receptor was kept rigid to the crystallographic position while all ligands were free to move and change conformations, and second, flexible docking calculations were run (Macromodel 9.0) where, the ligand and all aminoacids within 6.0 Å from the ligand were free to move and change conformations.

For rigid docking calculations, Glide 3.5 was used, having default parameters. For flexible docking calculations, no solvation model was employed; however, a distance-dependent dielectric “constant” of 4r was used with the OPLS2003 force field. 1000 steps of Monte Carlo/Low Mode (MC-LMOD) search were run. On each run the result structure was fully minimized using the TNCG algorithm. During the LMOD structural perturbation, and during the subsequent energy minimization, all residues within 6.0 Å from the ligand were allowed to move freely. During Monte Carlo structural perturbation, the ligand was randomly rotated and translated inside the binding cavity. The remaining residues were treated as “frozen atoms.” Unique conformations were stored only if they were within the lowest 50 kJ/mol.

The crystallographic water molecule located between, Arg394 and Glu354 (ERα) and the ligand phenolic moiety has been kept in all calculations. Previous studies have been shown that in the presence of this water molecule docking calculations can predict successfully crystallographic orientation of known ligands while its exclusion results to docking poses other than the crystallographic one (Fokialakis et al., 2004; Lambrinidis et al., 2006).

## Results

### Chemistry

#### Synthesis of phenyl esters

**Figure 2:**
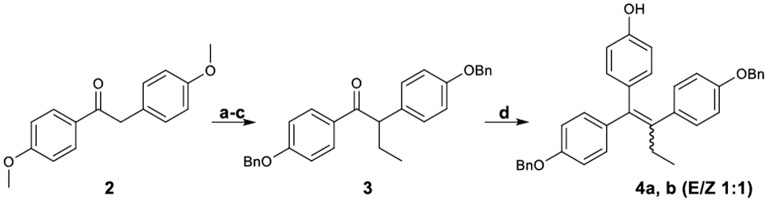
Synthetic Scheme 1: Reagents and conditions: (a) NaH, EtI, DMF, THF; (b) HBr, AcOH, 100°C; (c) K_2_CO_3_, BnBr, DMF, 60°C; (d) i. *n*-BuLi, BrC_6_H_4_OTBDMS, −78°C; ii. HCl.

Figure 2 demonstrates the synthesis of 1,2-bis-(4-benzyloxyphenyl)-1-(4-hydroxyphenyl)-1-butene (**4**), which was served as the key intermediate for the synthesis of the target carboxylate analogs. More specifically, desoxyanisoin **2** by alkylation, desmethylation and benzylation was transformed into ketone **3** in very good yield. Subsequently, reaction of ketone **3** with *tert*-butyl-dimethyl-phenoxy-silane lithium readily generated by treatment of (4-bromo-phenoxy)-tert-butyl-dimethyl-silane with 1 equivalent of *n*-butyllithium, yielded the corresponding carbinol, which under acidic conditions was simultaneously dehydrated and deprotected to provide compound **4** as a 1:1 mixture (E/Z, **4a/4b**) of diastereomers.

The diastereomeric ratio of these compounds was determined by separating the *E*,*Z* isomers using a semi-preparative HPLC Their structures were confirmed by NOE experiments, which distinguished the *E*-isomer considering the absence of a correlation among the protons of the ethyl group and the unprotected phenol protons. On the contrary, these correlations were always present for the *Z*-isomer. Additionally, the CH_2_ benzylic peaks discrimination is in accordance with a previously reported pattern (Detsi et al., 2002), since the triarylethylene *E*-isomer which was provided subsequently the desired *Z* isomer appeared more downfield (5.09, 5.01 ppm) as compared to those of the undesired isomer (4.99, 4.92 ppm).

**Figure 3:**
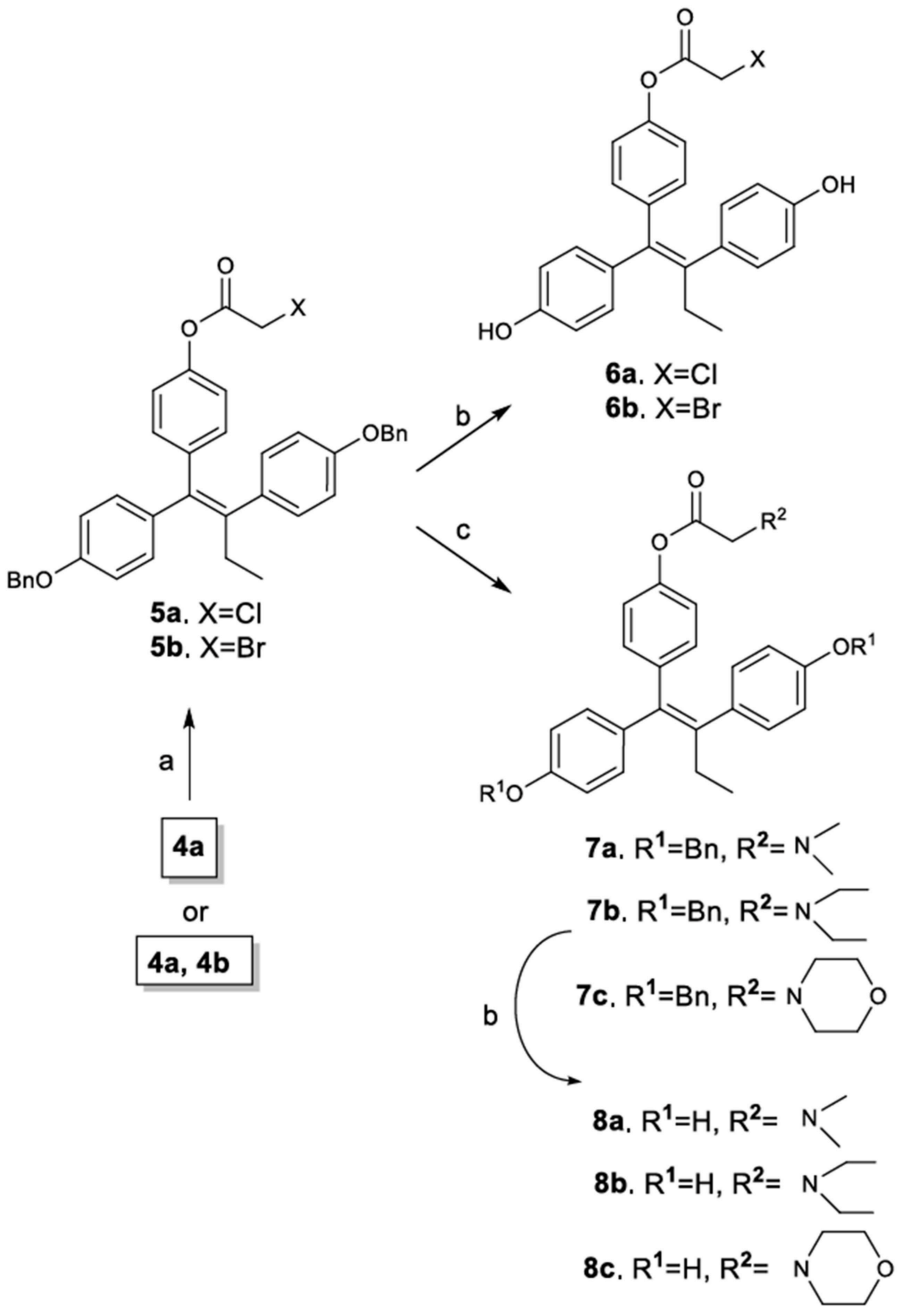
Synthetic Scheme 2: Reagents and conditions: (a) XCH_2_COX, pyridine, Et_2_O, 0–5°C; (b) H_2_, Pd/C, EtOAc; (c) Et_3_N, CH_3_CH_2_NHCH_2_CH_3_ or morpholine or CH_3_NHCH_3_, THF.

The synthetic route to novel TAM carboxylate analogs is illustrated in Figure 3. It must be noted however, that although the synthetic process can be accomplished using as substrate either the *E, Z* mixture of compounds **4a,b** or the pure *E* isomer **4a**, it is preferable the use of the mixture since the final products are obtained as mixture of diastereomers containing a prevailing amount of the desired *Z*-isomer (*Z*/*E* >11:1). More specifically, the esterification of phenol **4a** (or mixture of **4a**,**b**) provided the corresponding halogenated carboxylate derivatives **5a** and **5b** as 11:1 mixture of *Z/E* diastereomers (determined by ^1^H NMR and verified with HPLC). The amount of the desired *Z*-isomer was subsequently enriched by repetitive crystallizations with diethyl ether to achieve a final 20:1 mixture of *Z*/*E* diastereomers (determined by ^1^H NMR and HPLC). Compounds **5a** and **5b** were further reacted with diethylamine, morpholine and dimethylamine to afford the corresponding amines **7a**, **7b**, and **7**, which along with compounds **5a** and **5b** were debenzylated by catalytic hydrogenolysis to afford the target diphenols **6a**, **6b**, **8a**, **8b**, and **8c**. It is noticeable that the performance of the debenzylation reaction using as solvent the ethyl acetate proceeds without the hydrogenation of the sterically hindered double bond (Ruenitz et al., 1996; Detsi et al., 2002).

#### Preparation of acid-amides

**Figure 4:**
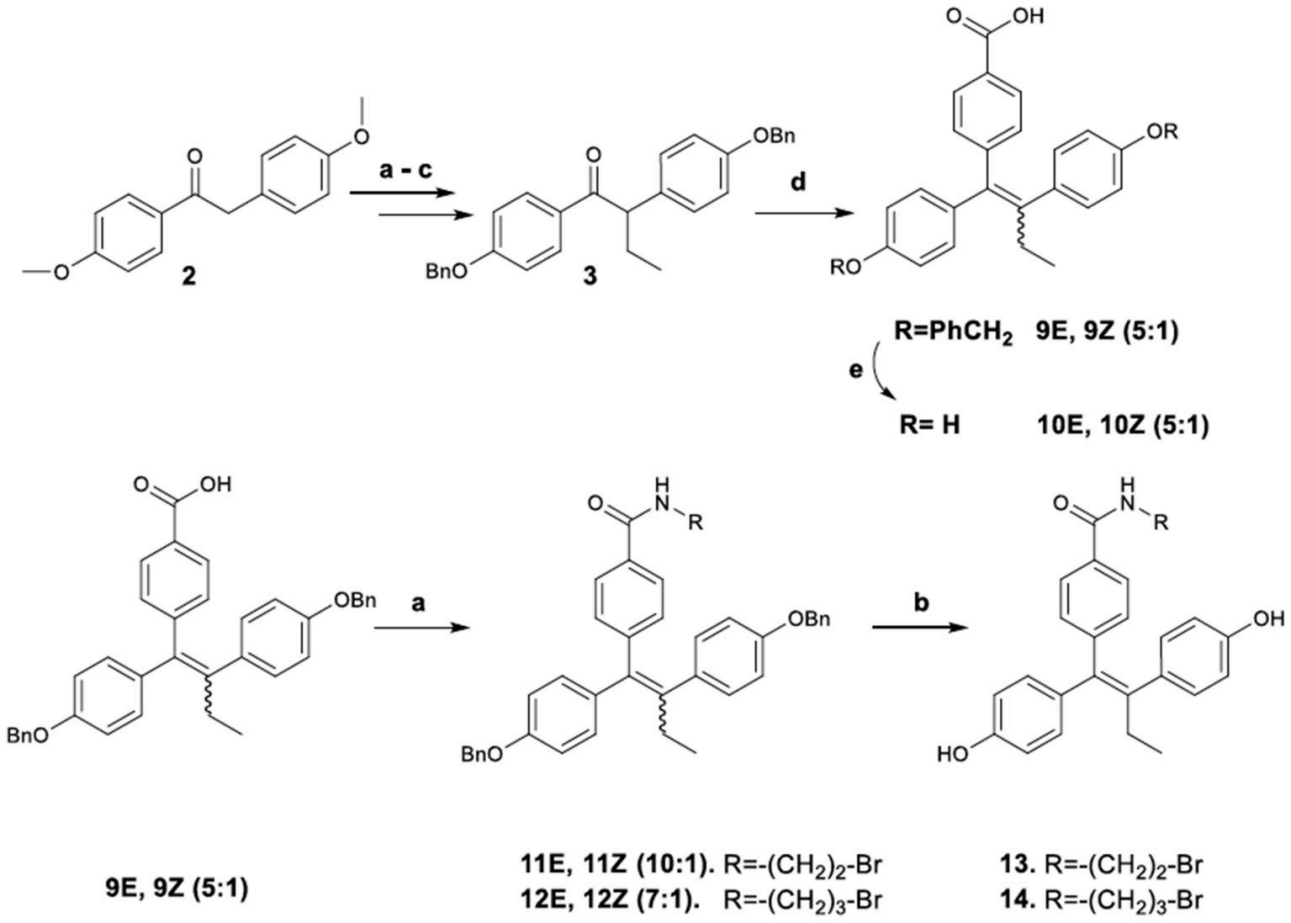
Synthetic Scheme 3: Reagents and Conditions (a) NaH, EtI, DMF, THF; (b) HBr, AcOH, 100 C; (c) K_2_CO_3_, BnBr, DMF, 60°C; (d) i. *n*-BuLi, BrC_6_H_4_COOH, THF, –78°C; ii. HCl; (e) H_2_, Pd/C, MeOH; (e) i. SOCl_2_, THF, 60°C; ii. RNH_2_, Et_3_N, DMF, 100°C; (f) H_2_, Pd/C, MeOH.

The synthetic pathway to acid-amide analogs of TAM from desoxyanisoin **2** substrate is illustrated in Figure 4. In particular, substrate **3** by alkylation, desmethylation and benzylation was efficiently transformed to ketone **3**, which was subsequently reacted with dilithiated 4-bromo benzoic acid (generated by treatment of 4-bromo-benzoic acid with 2 equiv of *n*-butyllithium) to yield the corresponding carbinol. The latter, under acidic conditions was simultaneously dehydrated to provide acid **9** as a mixture of *E*/*Z* diastereomers (**9*E/*9*Z*** 5:1 respectively). The diastereomeric ratio of these compounds was determined with the HPLC separation of the *E*,*Z* isomers. The NOE experiments distinguished the *Z*-isomer based on the correlation among the ethyl group protons and the protons of the benzoic acid. No similar correlation was observed for the *E*-isomer. Additionally, we noticed that the set of benzylic proton signals which correspond to each of the two isomers follow a similar pattern, regarding their shifts, in the ^1^H-NMR spectra. In this context, the OCH_2_ singlets for the isomer **9*Z*** show a downfield shift of Δ = 0.06–0.07 ppm as compared to the corresponding peaks of the *E* isomer.

Catalytic hydrogenolysis of the crude mixture provided the 4-[1,2-bis-(4-hydroxy-phenyl)-but-1-enyl]-benzoic acid **10** [previously reported in a patent containing symmetric triphenyl compounds (SMITHKLINE-BEECHAM-CORPORATION, 2007)^1^], as a mixture of diastereomers (**10*E***/**10*Z***, 5:1 respectively), which were separated by repetitive fractional crystallization from diethyl ether. Analytically pure samples of these compounds were obtained by semi–preparative HPLC.

On the other hand, a crude *E*/*Z* mixture of acid **9** was treated with 2-bromoethylamine and 3-bromopropylamine hydrobromates to provide amides **11**, **12** (as a mixture of enriched *E*/*Z* isomers, respectively in 10:1 and 7:1 proportions). The *E*-isomers predominance was again confirmed by spectroscopic data (^1^H NMR, NOE experiments). Finally, the amides were debenzylated to produce the target diphenols **13, 14**, which maintained the predominant *E* configuration. Analytical samples of all compounds were obtained by semi–preparative HPLC and used for their spectroscopic determination (^1^H NMR, NOE experiments) and their bioactivities evaluation.

### Binding to isolated human ERα and ERβ

The RBA values of the new TAM derivatives are summarized in Table 1. The phenyl ester analogs **6a,b** and **8a,b** demonstrated particularly high RBA values, indicative of their strong bind affinity to both ERα and ERβ, with a slight preference for the ERα subtype. Ester **8c** displayed lower binding affinity as compared to the other esters. Substitution of the ester group with an acid (compound **10)** and consecutive transformation to amides provided compounds, **13** and **14** displaying moderate to good RBAs as well. These binding affinity scores are thoroughly commented in next sections, in relation to docking calculations and conformational analysis results.

### Inhibition of E2 induction of proliferation of MCF-7 cells

**Figure 5:**
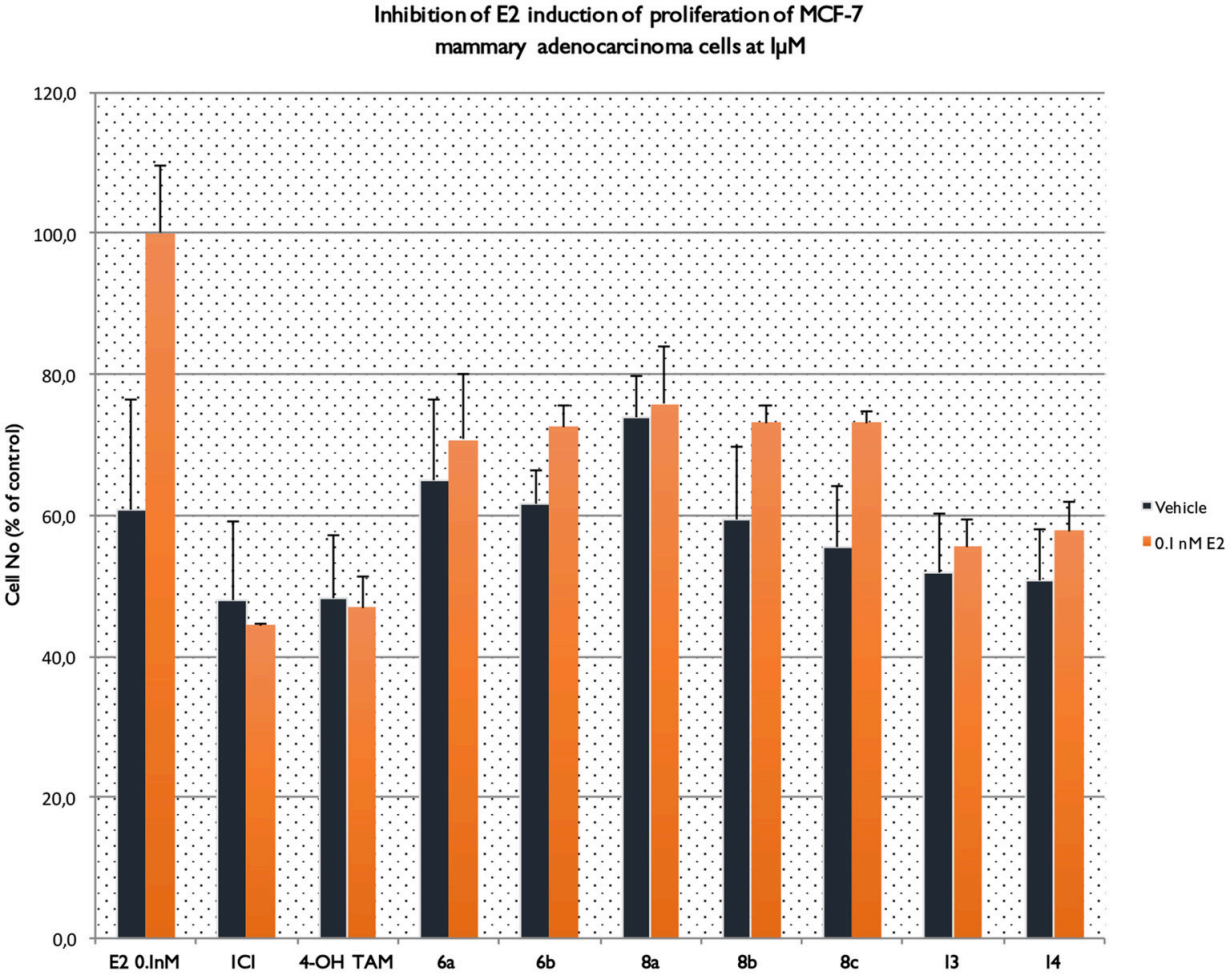
Inhibition of E2 induction of proliferation of MCF-7 cells.

One of the aims of this study was to investigate whether the novel TAM derivatives were able to inhibit the E2 induced proliferation of MCF-7 breast cancer cells (see Figure 5). In this context, it is apparent that phenyl esters **6a,b** and **8a–c**, display agonistic profile since they “failed” to inhibit E2 induction of proliferation of MCF-7 cells. On the contrary, amides **13** and **14** (especially **13**) at the same dose of 1 μM managed to substantially inhibit the respective proliferation, which to one extent underpins antagonistic profile.

### Theoretical calculation

In the presented work, most of the synthesized TAM analogs proved to be antagonists or partial agonists (see respective assay's results in **Figure 7**) exhibiting improved RBAs. In order to better understand the agonist activity of compounds **6a,b** and **8a–c** and the antagonist activity of compounds **13** and **14**, docking calculations were employed investigating their binding mode in different existing crystal structures of ERα with h12 oriented in the agonist or antagonist position. Two different structures of ERα (having h12 in agonist position) were used taking into account receptor's plasticity, one in the complex with DES (PDB entry 3ERD) and the other in complex with ortho-trifluoromethylphenylvinyl estradiol (EZT) where receptor's plasticity allows fitting of a relatively voluminous estradiol analog (PDB entry 2P15). Additionally, the crystal structure of LBD-ERα in complex with 4OH-TAM, having h12 in the antagonist position was used (PDB entry 3ERT).

### Conformational analysis

**Figure 6:**
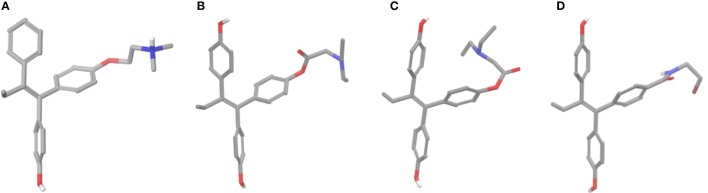
**(A)** Crystal structure of 4OH-TAM, **(B)** extended minimum structure of **8b, (C)** bend minimum structure of **8b**, and **(D)** global minimum structure of **13**.

Prior docking calculations, conformational analyses were performed for all analogs considering conformers of 4 kcal higher energy compared to the global minimum only. After 500 steps of conformational search two discrete conformers were found for all esters (Figures 6B,C). Global minimum structure had a conformation where the side chain had an extended orientation similar to crystal structure of 4OH-TAM which favors the formation of salt bridge with Asp351 (ERα) (Figures 6A,B). On the second structure, having 3–3.5 kcal/mol higher energy than global minimum, the side chain was oriented toward one of the phenyl groups. On the contrary, in the case of amides **13** and **14** the extended structure seems to predominate to other conformations that appear to be higher than 4 kcal to the global minimum. The calculated global minimum structures were used as initial structure for further docking calculations.

### Docking calculations

**Figure 7:**
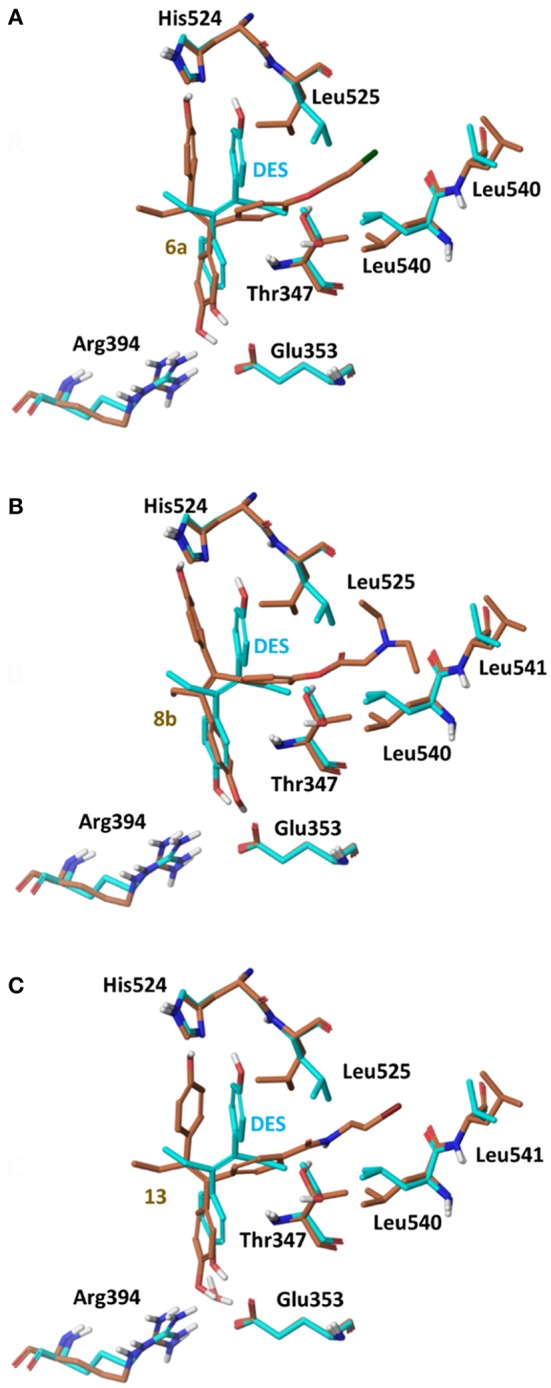
Superposition of Crystal Structure of ERα in complex with DES (cyan) and global minimum complex of ERα (PDB entry 3ERD) with **6a (A)**, **8b (B)**, and **13 (C)**.

Using the global minimum structures for all analogs rigid docking calculations were run using all 3 conformations of ERα (PDB entries 3ERD, 2P15 and 3ERT), to acquire initial complexes structures for further flexible docking calculations. On 3ERD all analogs were rejected as they could not fit inside binding pocket. A more detailed investigation of the binding pocket of PDB entry 3ERD showed that the main reason for binding rejection was the orientation of the side chain of residues Thr347 and Leu525 blocking the side chain of analogs **6** and **8** to be placed toward Helix 12 (Maximov et al., 2010). By rotating the side chains of Thr347 and Leu525 in an induced fit way rigid docking calculations were repeated all analogs were fitted well having the low energy (extended) orientation, (Figure 7).

**Figure 8:**
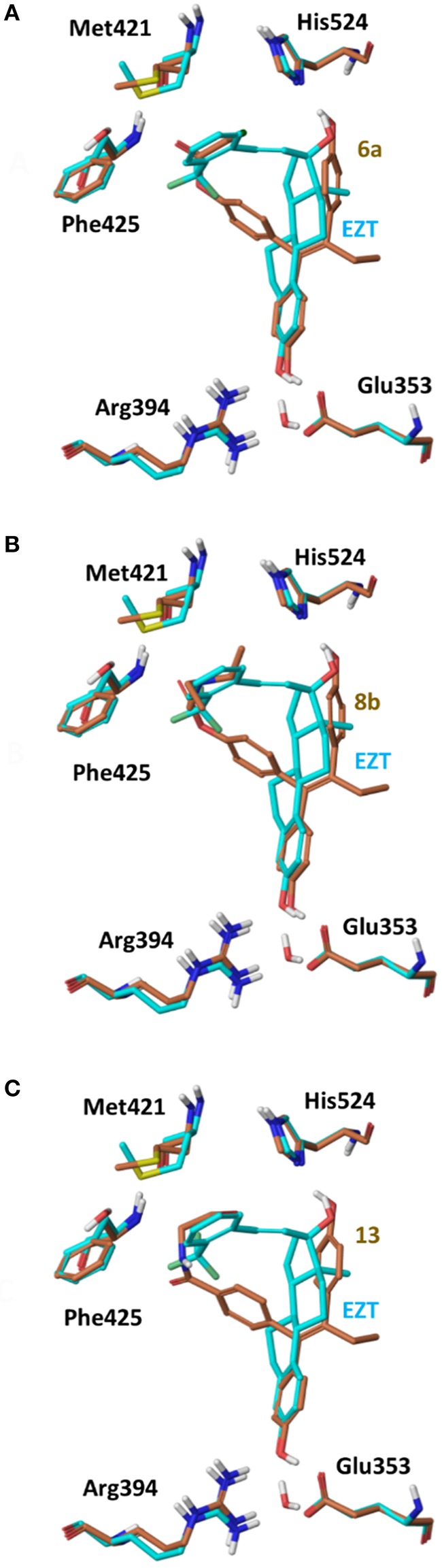
Superposition of Crystal Structure of ERα in complex with DES (cyan) and global minimum complex of ERα (PDB entry 2P15) with **6a (A)**, **8b (B)**, and **13 (C)**.

**Figure 9:**
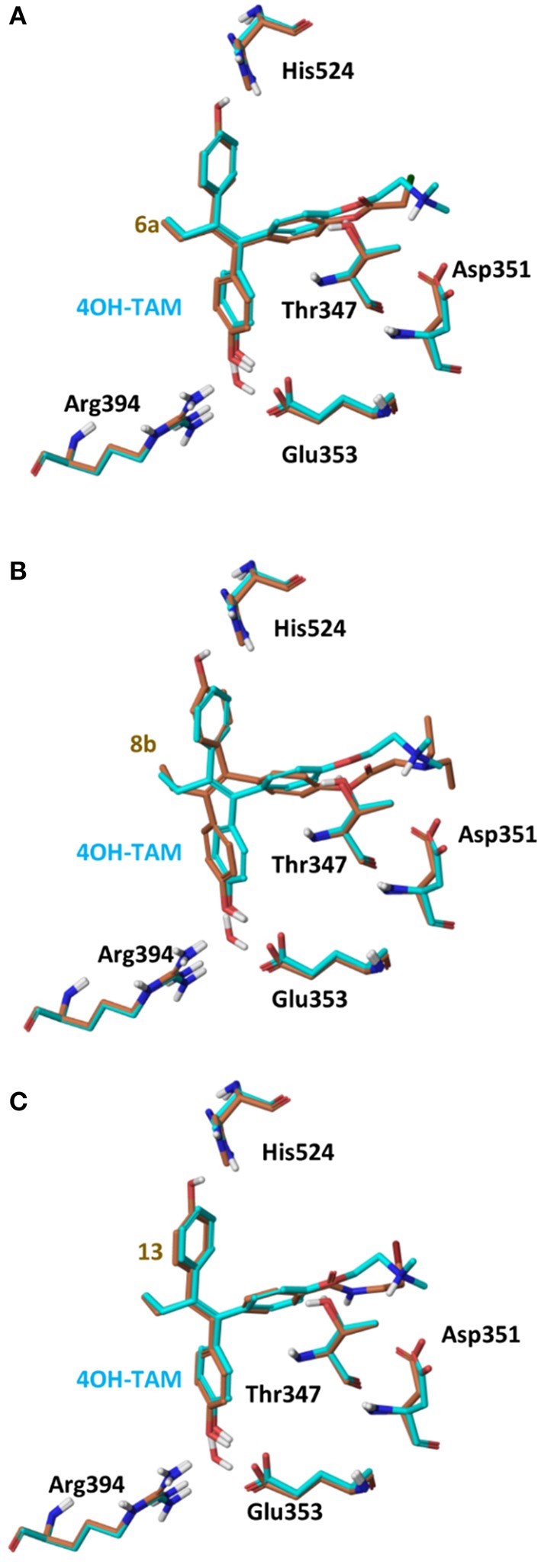
Superposition of Crystal Structure of ERα in complex with 4OH-TAM (cyan) and global minimum complex of ERα (PDB entry 2P15) with **6a (A)**, **8b (B)**, and **13 (C)**.

On 2P15 ERα structure the ligand binding domain displays structural plasticity accommodating, analogs **6a,b** and **8a–c** inside binding pocket. For compounds **13** and **14** (Figure 8) a high energy folded conformation was found to fit in the binding cavity which was not identified in the previous conformational search exciding the 4 kcal limit of accepted conformers. Finally, all compounds were able to fit in 3ERT cavity in an extended conformation similar to 4OH-TAM (Figure 9).

The calculated binding energies as reflected on the Glide scores are summarized in Table 2.

**Table 2:** Glide Scores (kcal/mol) of all studied compounds bound to ER agonist (2P15,3ERD) or ER antagonist conformation (3ERT).

**Compound**	**2P15**		**3ERD**		**3ERT**	
	**Neutral**	**Charged**	**Neutral**	**Charged**	**Neutral**	**Charged**
**6a**	−12.21		−12.06		−11.31	
**6b**	−12.01		−11.99		−10.97	
**8a**	−13.23	−9.30	−11.97	−12.86	−10.56	−11.87
**8b**	−12.54	−10.02	−11.97	**ND**	−10.75	−11.23
**8c**	−10.92	−10.17	−12.92	−12.82	−10.70	−11.80
**13**	−13.20		−12.90		−11.15	
**14**	−12.08		−13.28		−11.12	
4OH-TAM		−8.95		−10.98		−11.96

*Compounds **8a–c** studied in neutral or charged form of the side chain tertiary amine*.

*ND, No Valid pose found using Glide docking calculations*.

## Discussion

Synthetic pathways used for the construction of tamoxifen analogs (Kasiotis and Haroutounian, 2012) usually refer to the utilization of: (a) McMurry coupling, which affords a mixture of products consisting of the desired hetero-ketone coupling and the undesired homo-ketone coupling, or (b) organometallic nucleophiles for the construction of the carbon skeleton.

The presented synthesis was based on the rationale of constructing the triarylethylene framework and simultaneously keeping intact the double bond and the ethyl substituent, since a pertinent study (Lubczyk et al., 2002) has revealed that the SERM activity is enhanced for molecules containing substituents with two or three carbon chain as compared to those bearing either longer chains or only one carbon. Thus, the key intermediates **4a,b** and **9** were constructed using *n*-BuLi as the organolithium reagent, and then reactions were carried out efficiently building upon the free hydroxyl group or the acid moiety.

All synthesized compounds exhibited interesting biological activity. Compounds **6a,b** and **8a,b** displayed binding affinity to both ERα and ERβ higher than 4OH-TAM while compounds **13** and **14** have shown cellular antiestrogenic activity similar to 4OH-TAM and ICI182,780. Estrogen receptors are structurally characterized by the flexibility of the h12 C-terminal helix of the LBD, while a relative plasticity of the ligand-binding pocket has been demonstrated suggesting that ERs can interact with a wider array of pharmacophores still acting as agonists (Nettles et al., 2007).

The conformation of h12 has been proved to be crucial for estrogenic or antiestrogenic activity resulting in activation or repression of the estrogen target genes. SERMs are known to exhibit tissue-selective mixed agonist and antagonist activity which is dependent on the ER subtype and the induced distinct conformation of the liganded ER. It has however postulated that an equilibrium exist between different conformations of helix 12 which is dictated not only by the ligand but also by specific interactions between ER and other proteins such as co-activators and co-repressors that will finally result to gene expression (Gangloff et al., 2001).

In that terms, the final biological response is a multifactorial phenomenon depending both from ligand-protein and protein-protein interactions causing significant barriers in structure based rational drug design. Tamoxifen is the first classical SERM approved for clinical use exhibiting ER-antagonistic activity in breast tissue, while displays significant estrogenic activity in the skeletal and cardiovascular systems, liver and uterus. The active metabolite 4OH-TAM interacts in the ligand-binding pocket through the phenolic hydroxyl group forming strong hydrogen bonds (HB) with the Glu353 and Arg394. Although 4OH-TAM does not form a second HB with His524 in the distal part of the ligand-binding pocket (part of the classic estrogenic pharmacophore) is appended with a charged side chain protruding outward the LBD causing the repositioning of h12 into the hydrophobic ER surface where co-activators bind and thus disrupting the agonist activity. This side chain contains a basic positively charged amino group forming strong charge–charge interactions with Asp351. Our results show that compounds **6a** and **8a** display a mixed partial-agonist, partial antagonist activity. Figure 5 shows effects of estradiol, compounds **6a,b, 8a-c, 13, 14**, ICI182,780 and tamoxifen on the relative number of viable MCF-7 cells in the absence and presence of estradiol in the culture medium. Estradiol, **6a** and **8a** increased the number of viable cells relative to estrogen-free cells stimulating MCF-7 proliferation. Compounds **6b** and **8b** display no change compared to vehicle while **8c, 13**, and **14** reduces the number of viable cells. In the presence of estradiol all compounds antagonized hormonal increase with compounds **13** and **14** exhibiting a quite similar efficacy to 4OH-TAM (only 20% lower). Overall, compound **8a** displays partial agonist activity and low antagonist activity while compound **13** is characterized as antagonist.

Four of the ligands had an RBA >100 with **6a** displaying the highest affinity, 3-fold higher that tamoxifen. Among the amine substituted compounds **8a–c** the morpholino- substituted **8c** has the lowest affinity (RBA = 8.1). On the other hand, the amide compounds **13, 14** albeit of lower affinity than tamoxifen, display as aforementioned very similar biological profile on MCF-7 assays.

In order to generate useful structure activity relationships and get better insight on the ligand—ER induced final biological response we have carried out docking calculations of all synthesized ligands with three distinct ERα conformations the 3ERD structure to compare with the classic agonist diethylstilbestrol precursor of tamoxifen, the 2P15 an agonist conformation accommodating voluminous ligands and 3ERT to compare with tamoxifen.

The initial conformational analysis (Figure 6) suggested that ligands **6a,b** and **8a–c** are flexible enough to adopt folded conformations shaping structures that can be accommodated in the agonist ER forms. Indeed, all ligands can fit in the 2P15 ER conformation ligand pocket while they also fit after inducing minor conformational changes on the Thr347 and Leu525 side chains of 3erd. In this respect partial agonism of ligands **6a,b** and **8a,b** can be justified. Of course, all ligands can also behave as antagonists (as tamoxifen analogs) fully interacting with the ER antagonist conformation 3ERT. In all cases ligands display the typical interaction with Glu353 and Arg394 reinforced by a structural water molecule (observed in all ER crystal structures). Moreover the second phenolic hydroxyl group forms a HB with the distal His524 which can explain higher affinities of **6a,b** and **8a,b** compared to 4OH-TAM in which this interaction is missing. Interestingly in all theoretical structures of complexes with 3ERD and 3ERT ER conformations a HB between Thr347 hydroxyl group and side chain carbonyl seems possible to be formed (Figures 7, 9).

Tertiary amines **8a,b** have been considered in both states neutral and positively charged and in the latter case a salt bridge is formed with Asp351 as in 4OH-TAM. Calculated pKa values of compounds **8a–c** (using MarvinSketch v5.5.0.0 module as implemented on ChamAxon academic suite) were found to be 6.27, 6.82, and 4.23 respectively suggesting that at pH 7.4 all analogs are mainly neutral. However, a considerable population of compounds **8a** and **8b** at pH 7.4 is still charged and will form the salt bridge with Asp351 influencing positively the affinity toward ER. On the other hand, compound **8c** should be considered only neutral not interacting with Asp351. Together with the fact that more morpholino group is more rigid and voluminous can explain the difference in affinity in comparison to analogs **8a** and **8b**.

The number of the theoretical binding energies for each and every compound as exemplified by Glide scores (Table 2) reflects partly the complexity of the phenomenon to be addressed as we have considered only three ER conformations while at least 3 more exists in the deposited PDB structures. Moreover, one has to take into account the equilibrium with third part co-activators and co-repressors interacting with ER. Antagonists **13, 14**, and **8c** show lower theoretical binding energies with the 3ERT structure compared to 4OH-TAM in agreement with experimental results. It should be also taken into account that folded conformations of amides **13, 14** found to be of more than 4 kcal higher energy in the free ligand form thus the interaction with 2P15 although theoretically possible they should be precluded from appreciably impacting the estrogenic response. In the case of **6a** and **8a** partial agonism should prevail and the higher binding energies with both 2P15 and 3ERD agonist conformations should explain the higher RBA compared to 4OH-TAM and corresponding antagonist conformations. Finally, experimental RBA of weak antagonists **6b** and **8b** should be considered as an average of all different states in equilibrium and albeit the high theoretical affinities foreseen with conformation 2P15, the complexity challenges the limitations of theoretical models.

## Conclusions

The design and synthesis of novel tamoxifen derivatives unveiled analogs with pronounced binding affinity to Estrogen Receptors. Basic structural feature of the new compounds is the introduction of carbonyl group either in the form of carboxylate esters or as acid and amidic derivatives. Docking calculations supported the findings concerning the prominent binding affinities. Preliminary *in vitro* data in ER+ breast cancer cell line, along with the RBA values, positioned carboxylate esters as partial agonists, while amidic derivatives as antagonists.

More specifically compound **13** exhibits a comparable to 4OH-TAM profile in MCF-7 breast adenocarcinoma cells, however its relatively longer side chain than the tamoxifen corresponding one, exclude estrogenic biological activity albeit the diversity of the specific tissue environment. As such compound **13** is a promising analog challenging tamoxifen drawback such as agonistic activity in uterus and should be further investigated on animal models. Finally, one should consider the biological response of compounds **8a–c** which is modulated from partial agonist to weak antagonist according to the tertiary amine substituents and thus could be considered as probes in biological experiments. Additional testing with more human breast cancer cell lines (including ER-) is currently underway to further support the encouraging findings of this work and finally determine the role of estrogen receptor.

## Author contributions

SH outlined the research strategy and idea. KK carried out the synthetic reactions and complemented in the synthetic strategy. GL performed docking studies. EM complemented docking studies. NF conducted NMR experiments. ET contributed to the synthetic reactions. SH and KK drafted the manuscript. All authors read and approved the final manuscript.

### Conflict of interest statement

The authors declare that the research was conducted in the absence of any commercial or financial relationships that could be construed as a potential conflict of interest.

## Footnotes

^1^SmithKline-Beecham-Corporation, WO2007/62148 A2, 2007.
