# Supplementary material for: Novel Carbonyl Analogs of Tamoxifen: Design, Synthesis, and Biological Evaluation
Source: Front Chem. 2017 Sep 26;5:71. doi: 10.3389/fchem.2017.00071 (PMC5622936; doi:10.3389/fchem.2017.00071)
Supplement: Supplementary file 1 [file DataSheet1.docx]

Supplementary Material

Novel Carbonyl Analogues of Tamoxifen: Design, Synthesis, and Biological Evaluation

**Konstantinos M. Kasiotis,^1,*^ George Lambrinidis,^2^ Nikolas Fokialakis,^3^ Evangelia N. Tzanetou,^4†^ Emmanuel Mikros^2^ and Serkos A. Haroutounian^4,*^**

*** Correspondence:**

Corresponding Authors
Professor Dr. Serkos A. Haroutounian, [sehar@aua.gr](mailto:sehar@aua.gr)

Dr. Konstantinos M. Kasiotis, [K.Kasiotis@outlook.com](mailto:K.Kasiotis@outlook.com)

# Supplementary Data

**File 1.** Supplementary material 1

**File 2.** Supplementary material 2
