# Supplementary figures and images for: Novel Carbonyl Analogs of Tamoxifen: Design, Synthesis, and Biological Evaluation
Source: Front Chem. 2017 Sep 26;5:71. doi: 10.3389/fchem.2017.00071 (PMC5622936; doi:10.3389/fchem.2017.00071)

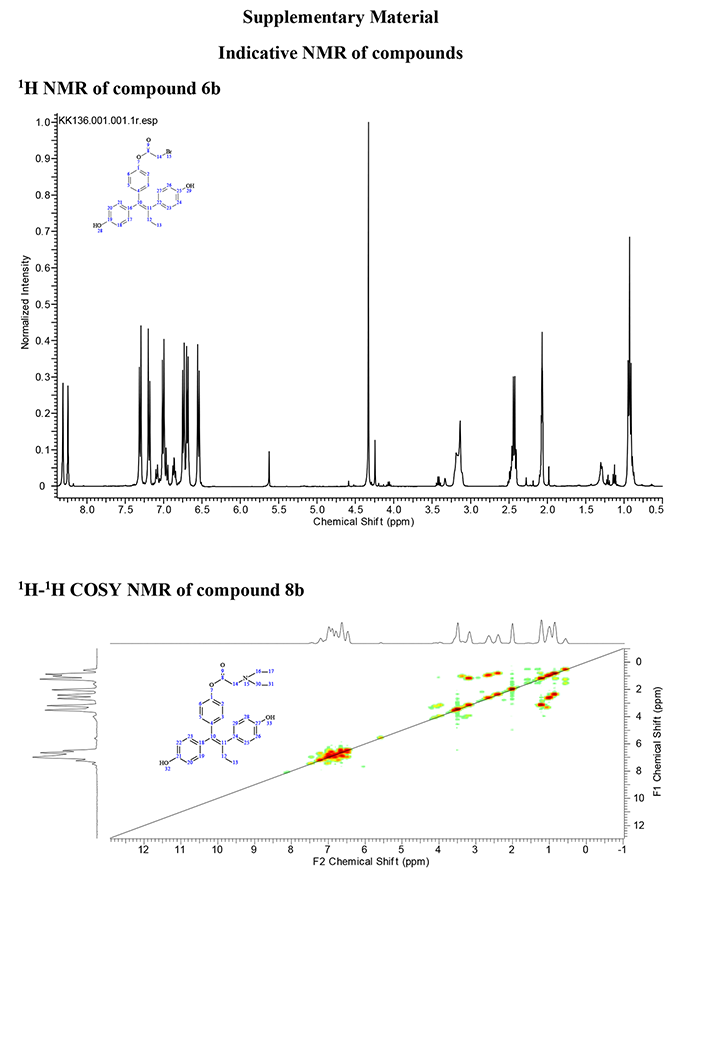

Supplement: Supplementary file 2 [file Image1.TIF]

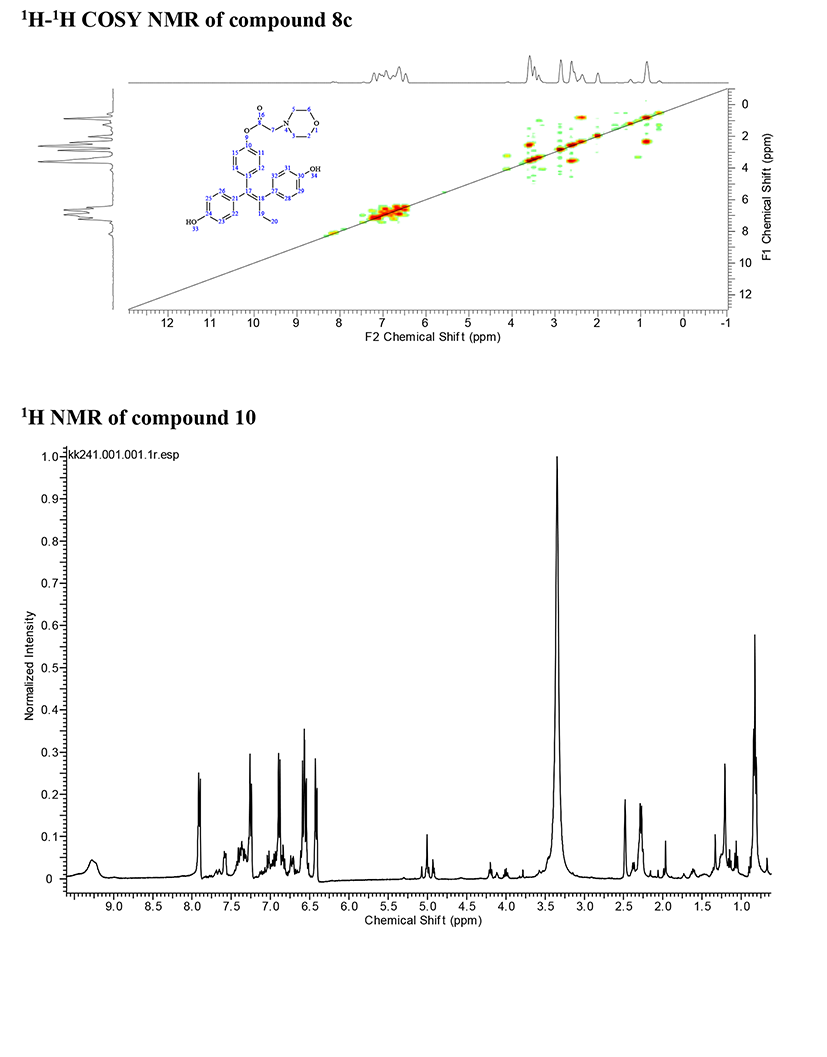

Supplement: Supplementary file 3 [file Image2.TIF]
